# Supplementary material for: Iridium-Functionalized Cellulose Microcrystals as a Novel Luminescent Biomaterial for Biocomposites
Source: Biomolecules. 2022 Aug 23;12(9):1165. doi: 10.3390/biom12091165 (PMC9496223; doi:10.3390/biom12091165)
Supplement: Supplementary file 1 [file biomolecules-12-01165-s001.zip › biomolecules-1813977-supplementary.pdf]

Iridium-functionalized cellulose microcrystals as a novel luminescent biomaterial for biocomposites

Mirko Maturi[a], Chiara Spanu[a], Andrea Baschieri[b], Mauro Comes Franchini[a], Erica Locatelli[a,] and Letizia Sambri[a,*]*

[a] Department of Industrial Chemistry "Toso Montanari", University of Bologna, Viale Risorgimento 4, 40136 Bologna, Italy.

[b] Institute for Organic Synthesis and Photoreactivity, National Research Council of Italy (ISOF-CNR), Via Gobetti 101, 40129 Bologna, Italy

SUPPORTING INFORMATION

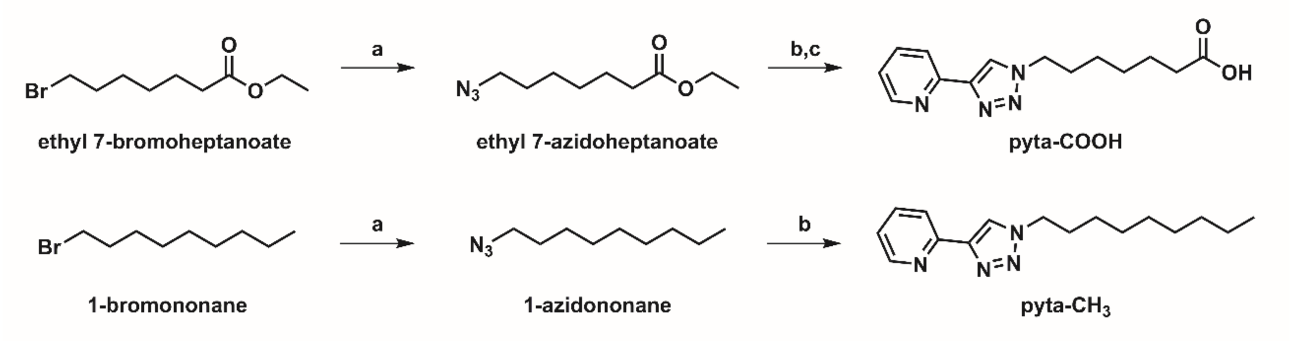

Figure S1. Synthesis of the pyridyl triazole ligands. Reaction conditions: a) NaN_3, DMSO, r.t., 24 h; b) 2-ethynylpyridine, $CuSO_4 \cdot 5\ H_2O$, Na-ascorbate, t-BuOH/H_2O 1:1, r.t., 24 h; c) KOH, DCM:EtOH:H_2O 8:3:1, r.t., 48 h.

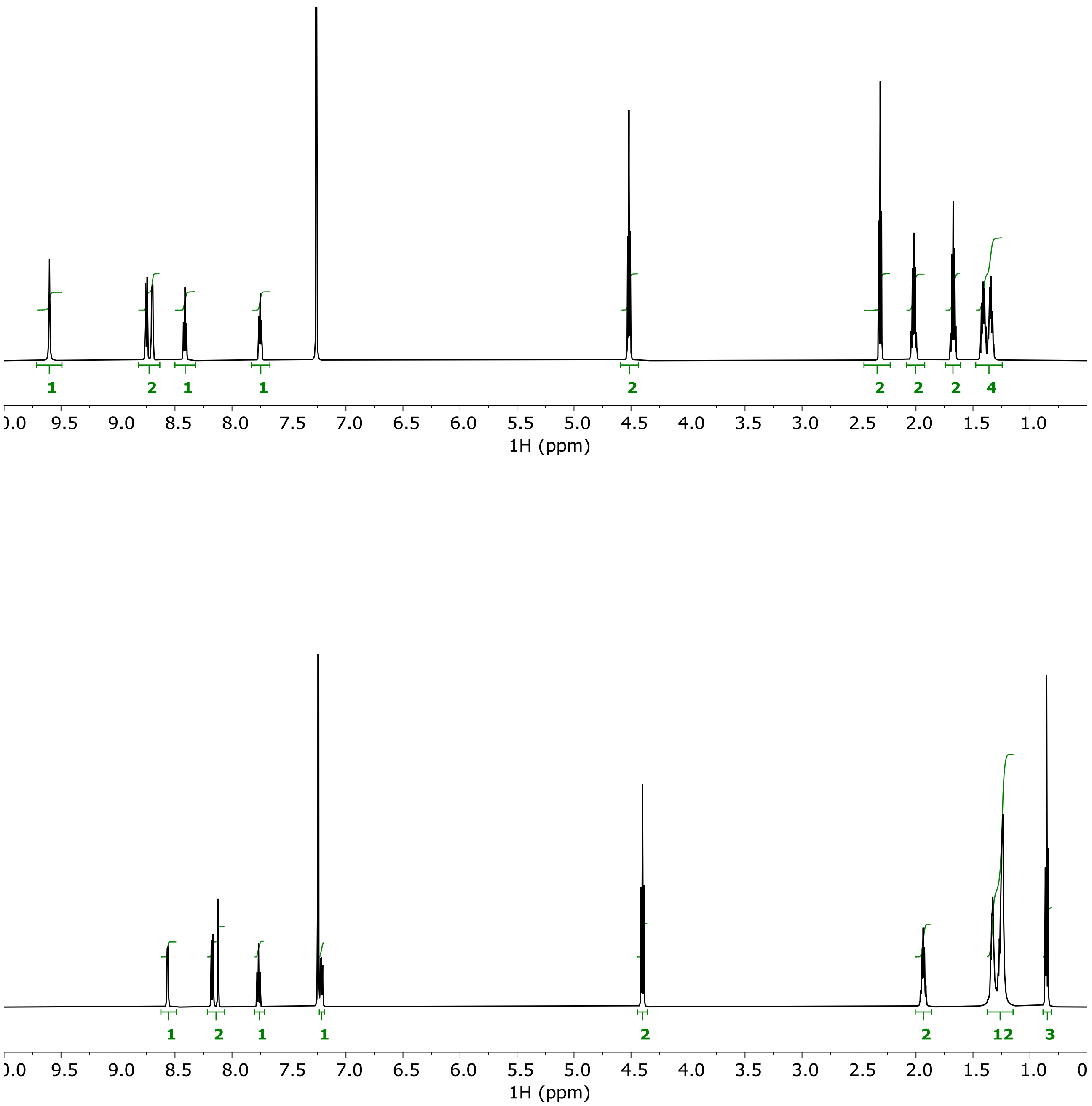

Figure S2. ^{1}H-NMR (600 MHz, chloroform-*d*) of pyta-COOH (top) and pyta-CH_3 (bottom).

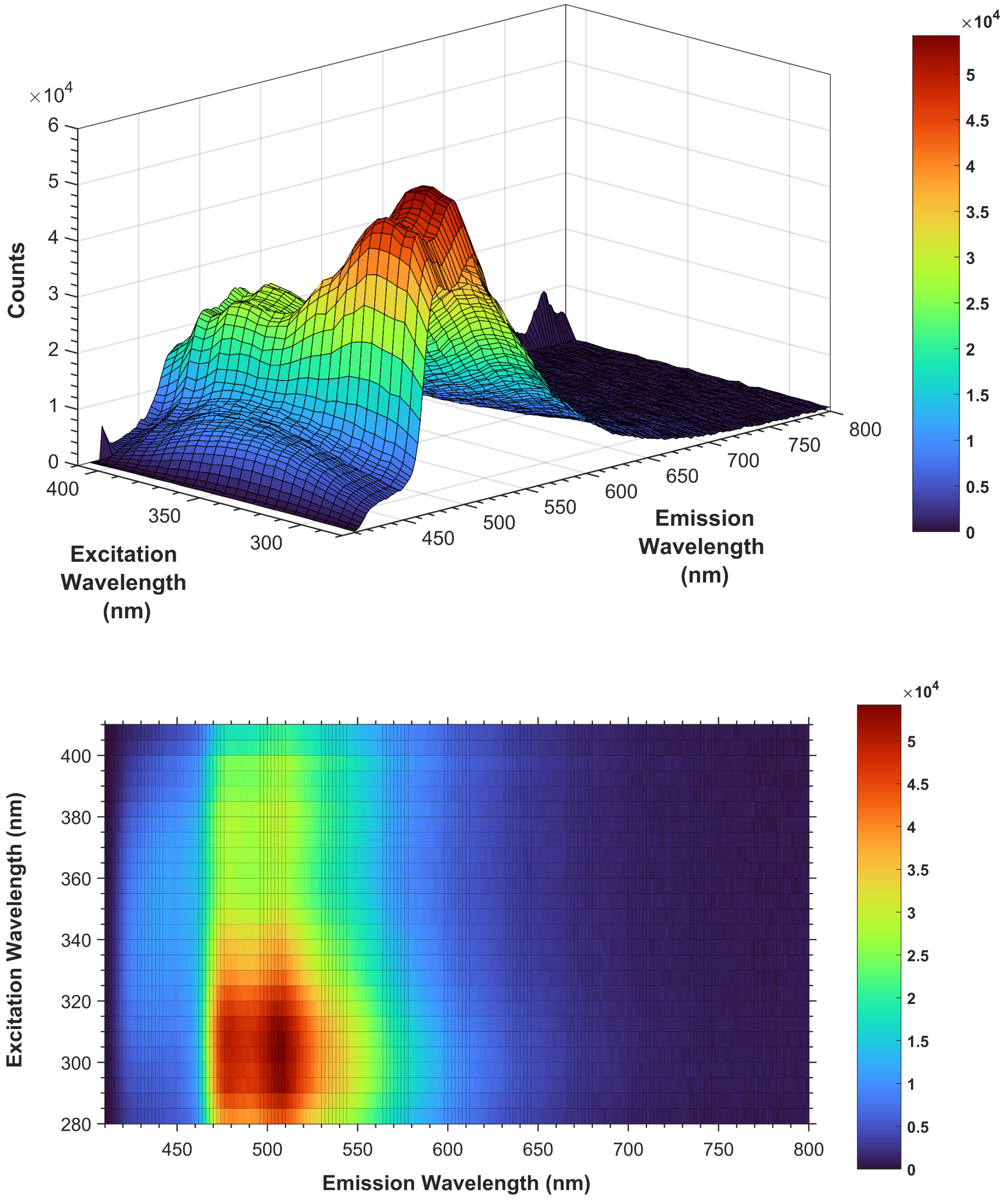

Figure S3. Emission maps of MCC-Ir. The same emission data are represented with a 3D surface plot (top) and with a colour-scale 2D image plot (bottom). Colour scale bars represent counts of the photodetector.

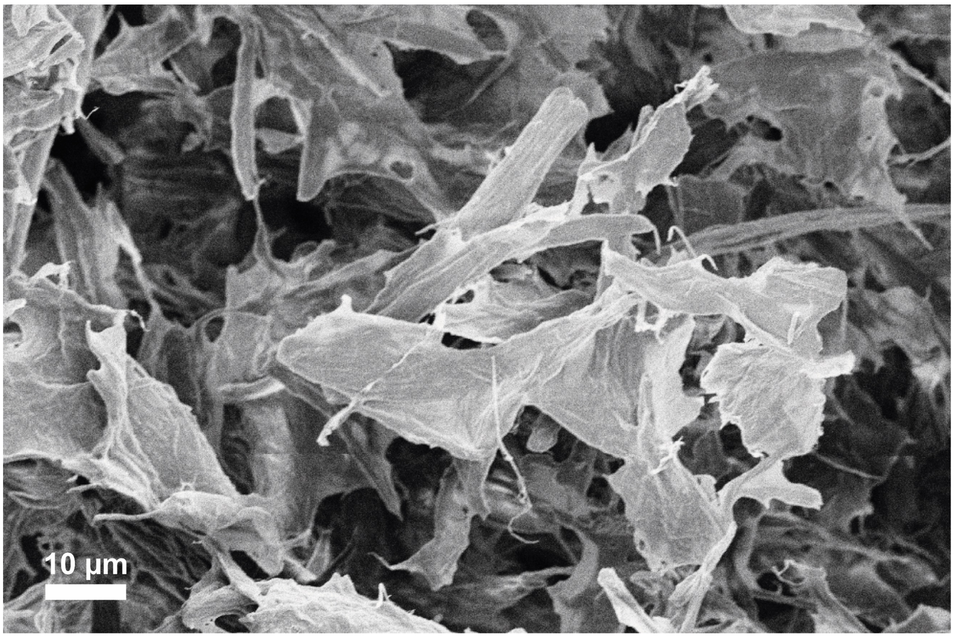

Figure S4. SEM image of MCC-OH

Photostability experiments

The double exponential decay represents the sum of two exponentially decreasing functions, each one being ruled by the corresponding decay constant k. Since the values of the decay constants are very different, the photobleaching decay is characterized by a fast initial decrease in the emission intensity, followed by a slower decay over prolonged exposures. The coefficient α, instead, relates to the relative percentage of fast vs. slow decay, revealing that the fast exponential decay is mainly limited to small radiant exposures ($\alpha \sim 10\%$ in both cases).

	α	k_1 $\left[\left(\frac{J}{cm^2}\right)^{-1}\right]$	k_2 $\left[\left(\frac{J}{cm^2}\right)^{-1}\right]$	**Goodness of fit (R^2)**
**MCC-Ir**	10.35%	$5.824 \cdot 10^{-3}$	$7.231 \cdot 10^{-5}$	0.9885
**[Ir(ppy)$_2$(pyta-COOH)]Cl in PMMA**	11.97%	$3.434 \cdot 10^{-3}$	$4.790 \cdot 10^{-5}$	0.9901

Table S1. Fitting parameters for the double exponential decay of phosphorescence intensity during the photostability experiment on MCC-Ir powder and [Ir(ppy)$_2$(pyta-COOH)]Cl in PMMA, according to **Equation 1**.
